# Decoding metabolic dysfunction in cancer: foundations for early detection and personalized therapeutics

**DOI:** 10.3389/fendo.2025.1693142

**Published:** 2025-11-24

**Authors:** Pradeep M. K. Nair, Ayyappan Palanisamy, Sekar Sivaranjani, Shanmugam Sudarshan, Sridhar Shubhakarini, Elangovan Karthika, Muniappan Devibala, Maruthanayagam Saranya, Thangavelu R., Saravanan P., Manickam Mahalingam, Janira Kumari, Karishma Silwal

**Affiliations:** 1 Department of Integrative Oncology, Mirakle Integrated Health Centre, Pollachi, India; 2 Department of Integrative Medicine, Regen Medica Wellness, Kuala Lumpur, Malaysia; 3 Department of Naturopathy, Sant Hirdaram Medical College of Naturopathy and Yogic Sciences, Bhopal, India

**Keywords:** metabolic markers, cancer risk, early detection, epigenetic regulation, integrative oncology

## Abstract

The global burden of cancer continues to rise despite significant advances in conventional oncology, underscoring the urgent need for novel approaches to prevention and early detection. While cancer has traditionally been regarded as a genetic disease, mounting evidence highlights the role of metabolic dysfunction as a precursor to malignant transformation. Altered glucose utilization, amino acid metabolism, lipid synthesis, mitochondrial function, and disrupted methylation pathways contribute to oxidative stress, epigenetic instability, immune evasion, and tumor initiation. This paper discusses key metabolic markers such as homocysteine, lactate dehydrogenase, HbA1c, insulin, cortisol, neutrophil-to-lymphocyte ratio, C-reactive protein, vitamin B12, parathyroid hormone, ionized calcium, estrogen and progesterone, and their potential as early indicators of cancer risk. Drawing on insights from integrative oncology practice, we highlight how metabolic markers can serve as both predictive and prognostic tools, complementing standard genetic and imaging diagnostics. Importantly, these markers should not be viewed in isolation but collectively, as they interact through overlapping biochemical pathways that foster tumorigenesis. Early identification of metabolic abnormalities may enable timely interventions to restore balance and mitigate cancer risk. However, cumulative and multicentric data are needed to validate their translational utility across diverse clinical settings.

## Introduction

The global burden of cancer is rising at an alarming rate, representing a leading cause of morbidity and mortality worldwide (1). Despite advances in therapeutic strategies including surgery, chemotherapy, radiotherapy, targeted therapy, and immunotherapy the prognosis for many cancers remains poor, particularly when diagnosed at advanced stages (2, 3). This underscores a critical need for improved strategies not only for treatment but also for early detection and effective prevention.

Historically, cancer has been viewed primarily as a genetic disease, driven by accumulated mutations that disrupt cellular processes such as proliferation, apoptosis, and DNA repair (4). However, emerging research highlights a deeper layer of complexity, revealing that metabolic dysregulation plays a central role in the initiation and progression of malignancies. Before genetic mutations become phenotypically evident, a cascade of metabolic alterations such as changes in glucose utilization, amino acid metabolism, lipid synthesis, and mitochondrial function can occur, creating a pro-oncogenic environment conducive to cellular transformation (5, 6).

These metabolic anomalies are not merely byproducts of cancerous transformation but may act as upstream regulators or enablers of tumorigenesis (7). Metabolic dysregulation may co-evolve with genetic mutations, suggesting that altered metabolic states can create a cellular environment conducive to DNA instability, oxidative stress, epigenetic changes, and immune evasion, factors that collectively increase the likelihood of malignant transformation (8, 9).

Identifying molecular markers associated with metabolic dysfunction holds significant promise for early cancer diagnosis, risk stratification, and prevention. These metabolism-associated biomarkers may be helpful to reveal individuals who are metabolically primed for malignant transformation, before conventional genetic or imaging diagnostics detect pathological changes. As early-warning signals, such markers could enable timely lifestyle or therapeutic interventions aimed at restoring metabolic balance and halting cancer progression. Studies also highlight the translational potential of such approaches. Recent mechanistic evidence has shown that hepatic transcriptional regulators serve as critical links between lipid metabolism and oncogenic signalling (10). In particular, the transcription factor KLF10 has been demonstrated to protect against metabolic dysfunction–associated steatohepatitis by activating HNF4α-dependent lipid and bile acid pathways. This finding underscores the role of KLF10 in connecting metabolic stress and inflammation with hepatocarcinogenic potential. Collectively, these insights suggest that targeting metabolic dysregulation may represent a promising strategy for cancer prevention and therapy.

Furthermore, understanding these early metabolic alterations may uncover novel biological pathways that drive tumor initiation, offering opportunities for targeted therapies that intercept cancer at its metabolic roots. By integrating metabolic profiling with genomics and proteomics, oncology can move toward a paradigm where prevention and early intervention are informed not only by genetic predisposition but also by dynamic metabolic signatures. This paper explores key metabolic markers that may serve as potential targets for early detection, prevention, and management of cancer.

## Ferritin

Serum ferritin, a well-established marker of chronic inflammation, is elevated in various cancers including myeloma, breast, pancreatic, ovarian, and lung cancers (11–15). Traditionally known as an iron-storage protein, ferritin also plays iron-independent roles in cancer progression by promoting tumor growth and chemoresistance, possibly through macrophage-driven inflammatory pathways (15, 16). Excess iron in the body generates free radicals through reactions like the Fenton reaction, leading to oxidative stress. This damages proteins, lipids, and DNA. Highly reactive hydroxyl radicals form 8-oxo-2′-deoxyguanosine, a mutagenic marker linked to point mutations and increased cancer risk, making ferritin it a key oxidative stress biomarker (17). A large Korean cohort found higher ferritin was linked to lower colorectal cancer risk (18**).** Therefore, monitoring ferritin levels is important for evaluating systemic inflammation, oxidative stress, and potential cancer risk or progression.

## Homocysteine

Elevated plasma homocysteine (Hcy) levels are common in cancer patients. Genetic variations in Hcy detoxification pathways, low folate levels, and Hcy’s role in cell proliferation and tumor progression suggest its potential as a cancer biomarker (19). Tumor cells secrete elevated levels of Hcy, which closely correlate with cell proliferation (20). Abnormal DNA methylation, especially promoter hypermethylation, silences tumor suppressor genes and drives cancer progression (21). Homocysteine, a key methylation cycle intermediate, reflects methylation imbalances and may indicate epigenetic dysregulation (22).Meta-analyses show high Hcy is associated with increased colorectal cancer risk and other malignancies, likely via disrupted DNA methylation (23).Elevated homocysteine levels thus serve as potential biomarkers for cancer risk, linking metabolic disruption to epigenetic alterations in tumor development and therapy resistance.

## Vitamin D

Vitamin D or 25-hydroxycholecalciferol (25(OH)D3) is considered as an important nutrient that regulates numerous cellular functions inside the body. Vitamin D exhibits anti-cancer properties by downregulating survivin and thymidylate synthase, enhancing chemotherapy sensitivity, and promoting cell differentiation. It modulates gene expression through the VDR pathway, affecting cell adhesion and proliferation (24). Epidemiological studies suggest a strong inverse relationship between vitamin D levels and the incidence of colorectal, breast, and pancreatic cancers (25, 26). Epigenetic regulation via methylation of VDR-related genes further links vitamin D to cancer pathophysiology (27). Low vitamin D levels are epidemiologically linked to higher incidence of several cancers (28).While confounders exist, vitamin D remains a promising adjunct in cancer care. Therefore, screening vitamin D levels can serve both as a preventive and prognostic tool in cancer.

## Lactate dehydrogenase

Lactate dehydrogenase (LDH) catalyzes the conversion of glucose to pyruvate under anaerobic conditions (29). The tumor microenvironment is often hypoxic, resulting in consistently elevated LDH levels in cancer patients. This elevation reflects an adaptive strategy by tumor cells to sustain energy and nutrient supply, facilitated by the overexpression of the hypoxia-inducible factor 1 (HIF-1) transcription factor (30, 31). LDH has been reported to play a significant role in carcinogenesis and tumor progression by contributing to cell proliferation, invasion, and angiogenesis (29).LDH also serves as one of the potential biomarkers of metabolic dysfunction (32), which is considered as one of the precursor for carcinogenesis (8).A large meta-analysis of solid tumors found high pre-treatment serum LDH strongly portends worse survival (33). Hence, early detection of LDH may serve a potential biomarker for early detection and prevention of cancer.

## Glycosylated hemoglobin

Cancer cells are hypermetabolic, with deranged energy metabolism due to underlying mitochondrial dysfunction (34). This altered metabolic state often overlaps with mechanisms seen in diabetes, including impaired glucose regulation. Diabetes and cancer share common pathological features such as hyperinsulinemia, hyperglycemia, chronic inflammation, and altered levels of endogenous hormones, making the occurrence of one disease a potential risk factor for the other (35). This further supports the concept of metabolic cancer screening using tools traditionally employed to assess glycemic control in diabetes. Elevated HbA1c levels, reflecting chronic hyperglycemia, have been associated with increased cancer risk and may serve as a potential biomarker for cancer prediction, especially in metabolically primed individuals (36, 37).

## Serum insulin

Chronic hyperinsulinemia, a key feature of insulin resistance, has been increasingly recognized as a significant contributor to cancer development and progression. Elevated insulin levels promote tumorigenesis through several interconnected mechanisms (38). Insulin acts directly as a mitogen, activating proliferative and anti-apoptotic signaling pathways. It also enhances the insulin-like growth factor-1 (IGF-1) axis by increasing IGF-1 production and decreasing the levels of its binding proteins (IGFBP-1 and IGFBP-2), thereby amplifying mitogenic and anti-apoptotic signals (38, 39). Additionally, hyperinsulinemia reduces sex hormone-binding globulin (SHBG) concentrations, leading to increased bioavailability of estrogen, which is implicated in hormone-sensitive cancers (38). Furthermore, insulin resistance is often accompanied by systemic inflammation and oxidative stress, both of which create a tumor-promoting microenvironment (35, 38). These multifaceted effects of insulin and insulin resistance establish a strong mechanistic link between metabolic dysfunction and cancer risk. This positions serum insulin as a key metabolic marker that may help predict cancer risk in individuals with or without underlying metabolic dysfunction.

## Cortisol

Cortisol, a glucocorticoid hormone, plays a central role in regulating the body’s physiological response to both endogenous and exogenous stressors (40). Considering stress as an independent risk factor for cancer, and given cortisol’s role in promoting immunosuppression and hyperglycemia (41, 42), both of which contribute to tumorigenesis, elevated cortisol levels may serve as an early metabolic marker for predicting cancer risk (40, 43). Numerous studies report an interconnection between cortisol, stress, and cancer, primarily mediated through the activation of the hypothalamic-pituitary-adrenal (HPA) axis. This activation triggers a cascade of neuroendocrine and immunomodulatory responses, promoting a proinflammatory state and weakening the immune system. As a result, cancer cells may escape immune surveillance, facilitating tumor development and progression (44, 45).Hence monitoring cortisol levels can serve as a useful biomarker in cancer prediction and prognosis.

## Alpha-hydroxybutyrate dehydrogenase

Serum α-HBDH an isoenzyme of LDH is considered as a valuable prognostic marker in many cancers, especially lung, testicular and hematological cancer (46–48). Earlier studies also suggest the use of α-HBDH along with LDH and other cancer as a valuable diagnostic tool. α-HBDH is often overexpressed in malignant cells and contributes to elevated lactate production, a key feature of the tumor microenvironment. This excess lactate facilitates cancer cell proliferation, angiogenesis, and immune evasion (47, 49). Because α-HBDH levels correlate with tumor burden, aggressiveness, and hypoxia-driven metabolic shifts, it holds promise not only as a prognostic marker indicating disease severity and progression but also as an early indicator of metabolic dysfunction that may precede chronic diseases, including cancer.

## Neutrophil to lymphocyte ratio

An increased neutrophil-to-lymphocyte ratio (NLR), a marker of systemic inflammation, is increasingly recognized as a prognostic indicator in malignancy (50).Neutrophils, the most abundant subset of white blood cells, are perceived to contribute to cancer pathogenesis through their immunosuppressive and pro-tumorigenic properties (51). Furthermore, platelets, neutrophils, and lymphocytes are integral components of the tumor microenvironment and play a crucial role in promoting tumor cell proliferation and metastasis (52).Neutrophilia, often observed in cancer-related chronic inflammation, contributes to tumor progression through cytokine production, immune suppression, and metastasis promotion (53, 54). Simultaneously, a decline in lymphocytes weakens adaptive immunity, which is more predominant in cancers. NLR reflects this imbalance, capturing both the tumor-promoting impact of elevated neutrophils and the compromised antitumor defense due to reduced lymphocytes (54). Thus, NLR serves as a cost-effective and easily accessible marker for cancer detection and prognosis, reflecting the underlying inflammatory and immune status of the patient.

## C-reactive protein

CRP, one of the most reliable inflammatory marker is often elevated in many conditions including cancer. Very high CRP levels are associated with advanced, poorly responding metastatic cancers (55).CRP exists in two main forms: pentameric CRP (pCRP), the anti-inflammatory circulating form, and monomeric CRP (mCRP), which emerges during inflammation and is pro-inflammatory. The dissociation of pCRP into mCRP in response to tissue damage triggers immune activation, contributing to disease processes like endothelial dysfunction and leukocyte recruitment (56).Inflammation is considered as one of the hallmark of cancer and chronic inflammation has shown to promote pro-tumorigenic process that triggers rapid cell prolifereation (57). One review reported individuals with elevated CRP levels had a higher overall risk of developing cancer compared to those with lower levels. Site-specific meta-analyses also show that elevated CRP is significantly associated with increased risks of breast, colorectal, lung, and other cancers (58). Therefore, CRP being a sensitive marker for inflammation and tissue damage can be used both as diagnostic and prognostic markers for cancers.

## Vitamin B12

Vitamin B12 is an essential nutrient for cell division, playing a critical role in physiological functions and methylation pathways that contribute to DNA synthesis and stabilization (59). Higher level of vitamin B12 is commonly associated liver diseases, autoimmune disorders, renal diseases and infections (60). However, an imbalance in vitamin B12 levels can lead to DNA instability, triggering a cascade of genetic mutations. Studies also suggests prevalence of increased vitamin B12 levels (> 800 pmol/L) in case of solid cancers and hematological malignancies (60–62).Elevated vitamin B12 levels have been positively associated with an increased risk of several cancers, including those of the lung, pancreas, and liver, as well as certain myeloid-related hematological malignancies (63). Alternatively, deficient levels of vitamin B12 and other B vitamins can impair the methylation cycle, leading to hyperhomocysteinemia. This disruption compromises DNA methylation and stability, increasing the risk of genetic mutations and contributing to cancer initiation and progression (64). This highlights the potential value of monitoring vitamin B12 levels not only for early cancer detection but also as a prognostic indicator.

## Insulin-like growth factor 1

IGF-1 is a growth-promoting hormone that links nutrition and growth signaling. GF-1 has potent insulin-like metabolic effects, increasing cellular glucose uptake and favoring glycolysis (65). In tumors, IGF-1 signaling promotes the Warburg effect by enhancing glycolytic ATP production and supplying intermediates for anabolic growth. A large UK Biobank cohort study (n = 412,645; median follow-up 7.2 years) found that elevated circulating IGF-1 levels were associated with an increased overall cancer risk (66). Specifically, higher IGF-1 levels predicted significantly greater risks for breast, prostate, colorectal, melanoma, kidney, and thyroid cancers, while being inversely associated with the risks of lung, ovarian, head and neck, and liver cancers (66). Another prospective study from the EPIC-Heidelberg cohort reported a U-shaped association between IGF-1 levels and mortality, with both low and high IGF-1 linked to increased risks of cancer (67).IGF-1 and its isoforms IGF-1Ea, IGF-1Eb, and IGF-1Ec play critical roles in breast cancer by promoting proliferation, epithelial-to-mesenchymal transition (EMT), and metastasis via IGF-1 receptor (IGF-1R)-mediated signaling pathways (68). Taken together, these findings underscore IGF-1 as a metabolic onco-promoter and may serve as a potential biomarker for cancer risk assessment, screening, and prognosis.

## Lipoprotein(a)

Lp(a) is an LDL-like lipoprotein with apolipoprotein(a), known mainly for atherothrombotic risk. In a Japanese prospective cohort individuals with low Lp(a) (<80 mg/L) had about a 1.5-fold higher risk of cancer mortality than those with higher levels (69).This suggests low Lp(a) might be a marker of reduced anti-tumor defense. On the other hand, studies also show high Lp(a) (especially combined with high fibrinogen) can also portend cancer mortality (70).Though the mechanism is unclear Lp(a) these prospective observations illustrate that dysregulated lipid markers like Lp(a) intersect with tumorigenesis and aberrant levels (low or high) may warrant cancer surveillance in context of other risk factors.

## Immunoglobulin E

IgE is known for allergy and parasitic immunity. Recent studies indicate IgE also participates in anti-tumor immune surveillance. An EAACI task force review highlights that ultra-low or absent IgE is linked to higher malignancy rates. For instance, in the largest pediatric study to date (n=6,821), children with IgE deficiency had a dramatically higher odds of previous cancer (71). Most of these were hematologic cancers. In adult cohorts the picture is more nuanced in A 7 years follow up study among 37,747 individuals showed high IgE protective against chronic lymphocytic leukemia and possibly multiple myeloma (72). In summary, longitudinal data suggest that very low IgE (immunodeficiency) may flag impaired tumor surveillance, whereas higher IgE tends to correlate with lower risk of certain cancers (72, 73). Thus, measuring total IgE and noting abnormally low values could serve as a biomarker for elevated cancer risk, opposite to conventional inflammatory markers.

## Thyroid profile (TSH, T3, T4)

Thyroid hormones (T3, T4) set basal metabolic rate, impacting cancer growth. Abnormal thyroid function tests can signal altered cancer risk, for instance, subclinical hyperthyroidism has been linked to higher breast, lung, and prostate cancer rates (via increased angiogenesis and cell turnover), whereas overt hypothyroidism often confers relative protection. (74) Multiple prospective studies show that a hyperthyroid profile (high T3/T4, low TSH) generally raises cancer risk, whereas hypothyroidism is often protective. In the Rotterdam Study (n=10,318), higher baseline free T4 was significantly associated with incident cancers: each unit increase in FT4 predicted a 42% higher risk of any solid tumor and especially elevated risks of lung and cancer (75). Similarly, in an Australian 20-year cohort study, lower TSH and higher FT4 levels were associated with an increased risk of prostate cancer. Elevated TSH appeared protective, while higher FT4 was linked to greater risk (76). In contrast, a large Taiwanese cohort found that women with chronic hyperthyroidism actually had lower incidence of gynecologic cancers, whereas hypothyroid women had slightly higher risk (77)suggesting some context-specific differences. Overall, it is essential to maintain thyroid hormone levels within physiological homeostasis. Both hyper- and hypothyroid states may influence cancer risk and progression through metabolic and proliferative pathways and thyroid dysfunction may also serve as a potential biomarker for early detection or cancer prognosis.

## Gamma-glutamyl transferase

GGT is a liver enzyme involved in glutathione metabolism and antioxidant balance. It is a marker of hepatic injury, alcohol use, and metabolic syndrome. Elevated serum GGT is associated with diabetes, obesity, and chronic inflammation all known cancer risk factors (78). GGT may promote tumorigenesis by mitigating oxidative stress in cancer cells and supplying glutamate for glutathione synthesis. Clinically, persistently raised GGT should prompt evaluation for malignancy or metabolic syndrome. For example, one large cohort found that individuals with recurrently high GGT (top quartile) had significantly increased risk of respiratory cancers (78).A recent meta-analysis pooled 12 studies on gastrointestinal (GI) cancers: those in the highest GGT quartile had a 69% higher risk of GI cancer than the lowest quartile (79).This effect was strongest for colorectal and liver cancers, which showed significant trends across all GGT quartiles. ^81^ In the UK Biobank, higher serum GGT levels were associated with an increased risk of pancreatic cancer (80). Thus GGT serves as a metabolic–oxidative stress biomarker linking lifestyle/metabolic disorders to cancer risk.

## Parathyroid hormone

PTH regulates calcium/phosphate homeostasis. Many tumors (breast, lung, kidney, squamous carcinomas) secrete PTHrP, which mimics PTH to drive bone resorption and hypercalcemia (81).A recent meta-analysis found that PHPT patients have a 28% higher overall cancer risk, with papillary thyroid and breast cancers being the most common (82).The mechanism may involve PTH/PTHrP’s effects on cell proliferation and altered vitamin D/calcium metabolism. These findings indicate that dysregulated PTH/PTHrP-calcium signaling is a key metabolic hallmark of cancer, and persistent PTH elevation can serve as a biomarker of occult malignancy. Measuring PTH is therefore part of evaluating cancer-associated hypercalcemia. Furthermore, assessing parathyroid hormone (PTH) levels provides an indirect yet reliable measure of vitamin D status, as PTH maintains an inverse relationship with circulating vitamin D. Monitoring this balance can guide appropriate supplementation strategies and help ensure optimal physiological outcomes.

## Ionized calcium

Ionized calcium is the biologically active calcium fraction in blood. Hypercalcemia is a hallmark of advanced malignancy: about 20–30% of cancer patients eventually develop hypercalcemia (81). Mechanistically, tumor cells (e.g. lung, breast, myeloma) either secrete PTHrP or stimulate local osteoclasts, driving up ionized calcium (3). From a biomarker standpoint, an elevated corrected or ionized calcium level (especially with low true PTH) in a patient should raise suspicion for underlying malignancy. Thus, routine monitoring of calcium (and PTH/PTHrP) in cancer patients helps detect metabolic derangements early and guides interventions (hydration, bisphosphonates, etc.) to mitigate complications. (3) Similarly, ionized calcium levels reflect the biologically active fraction of circulating calcium and are closely regulated by vitamin D status. Monitoring ionized calcium alongside vitamin D offers valuable insight into calcium homeostasis, guiding supplementation and potentially highlighting metabolic imbalances linked to disease risk.

## Estrogen

Estrogens are steroid hormones (mainly 17β-estradiol and estrone) derived from cholesterol that drive proliferation in hormone-sensitive tissues. Elevated circulating estrogens are a well‐established risk factor for breast and reproductive cancers. Epidemiological studies in postmenopausal women show a 2–3 times higher breast cancer risk with high blood estradiol levels (83). In obesity and metabolic syndrome, aromatization of androgens in adipose tissue and lower sex hormone–binding globulin (SHBG) raise bioavailable estrogen (83), linking metabolic dysfunction to hormone-driven tumorigenesis. Numerous longitudinal studies link high estrogen levels to cancer. For example, pooled analyses of prospective cohorts (EHBCCG/EPIC) found that postmenopausal women in the highest quintile of estradiol had roughly 2 times the breast cancer risk of those in the lowest quintile (84). Similarly, endometrial and ovarian tissues exposed to estrogen also show elevated tumorigenesis (e.g. menopausal estrogen therapy increases endometrial and ovarian cancer risk) (85). Moreover, aberrant estrogen metabolism generates catechol and quinone metabolites that form DNA-damaging adducts (83). High levels of estrogen–DNA adducts have been detected in women with breast, ovarian and even thyroid cancer (and in men with prostate cancer) (86).Thus, both elevated estrogen levels and dysregulated estrogen metabolism serve as important biomarkers and mechanistic contributors to hormone-driven cancers.

## Progesterone

Progesterone (P4) is a C21 steroid hormone produced after ovulation by the corpus luteum of the ovary and, during pregnancy, by the placenta (with smaller amounts from the adrenal cortex). If progesterone is low or absent (for example, in anovulatory cycles or estrogen-only therapy), estrogen remains “unopposed,” leading to prolonged endometrial proliferation and high cancer risk (87). Excessive progesterone is again considered a risk factor. In the Women’s Health Initiative (WHI) trial, postmenopausal women receiving estrogen plus medroxyprogesterone had about a 24–26% higher incidence of invasive breast cancer than placebo whereas women on estrogen alone actually had slightly lower breast cancer rates (88). Animal and human studies show that progesterone (especially together with estrogen) robustly stimulates mammary epithelial proliferation (88). In summary, both extremes of progesterone exposure, i.e. too low (unopposed estrogen) or chronically high exogenous progestin can influence hormone-sensitive cancer development (87, 89).Hence, assessing progesterone levels alongside estrogen is crucial for identifying individuals at risk for hormone-sensitive malignancies.

**Supplementary File 1** presents a comprehensive overview of metabolic biomarkers and their oncogenic potential. The data are organized under the following categories: biomarker, normal range, altered level and associated cancer risk, highest level of evidence supporting the association, and summary of findings from the literature.

## Discussion

Cancer as a metabolic disease is gaining attention with the growing interest in precision medicine (90).While cancer is highly regarded as an outcome of genetic mutations happening within the patient, emerging evidence suggests the role of metabolic dysfunction as a predecessor of genetic abberrations (8). Despite remarkable progress in the standard of care for cancer management, the global burden of cancer and related mortality continues to rise each year (1), underscoring the urgent need for additional strategies in both prevention and treatment.

In the context of cancer prevention, early screening and detection continue to serve as foundational strategies for reducing incidence and improving outcomes. The present paper contributes an additional perspective by emphasizing the role of detecting early metabolic dysfunctions, that may precede genomic instability and tumorigenic mutations (6). Incorporating such metabolic markers into screening frameworks could refine risk assessment models, facilitate targeted surveillance of at-risk populations, and support timely preventive interventions. However, large-scale, multi-centric data are required to validate their predictive value and ensure their applicability across diverse clinical settings. In line with emerging evidence from molecular biomarker research in colorectal cancer (91), systematic screening of metabolic biomarkers may similarly offer a minimally invasive and cost-effective approach for early cancer detection, enabling identification of at-risk individuals before clinical onset and complementing existing genomic, proteomic, and transcriptomic assays.

**Figure 1:**
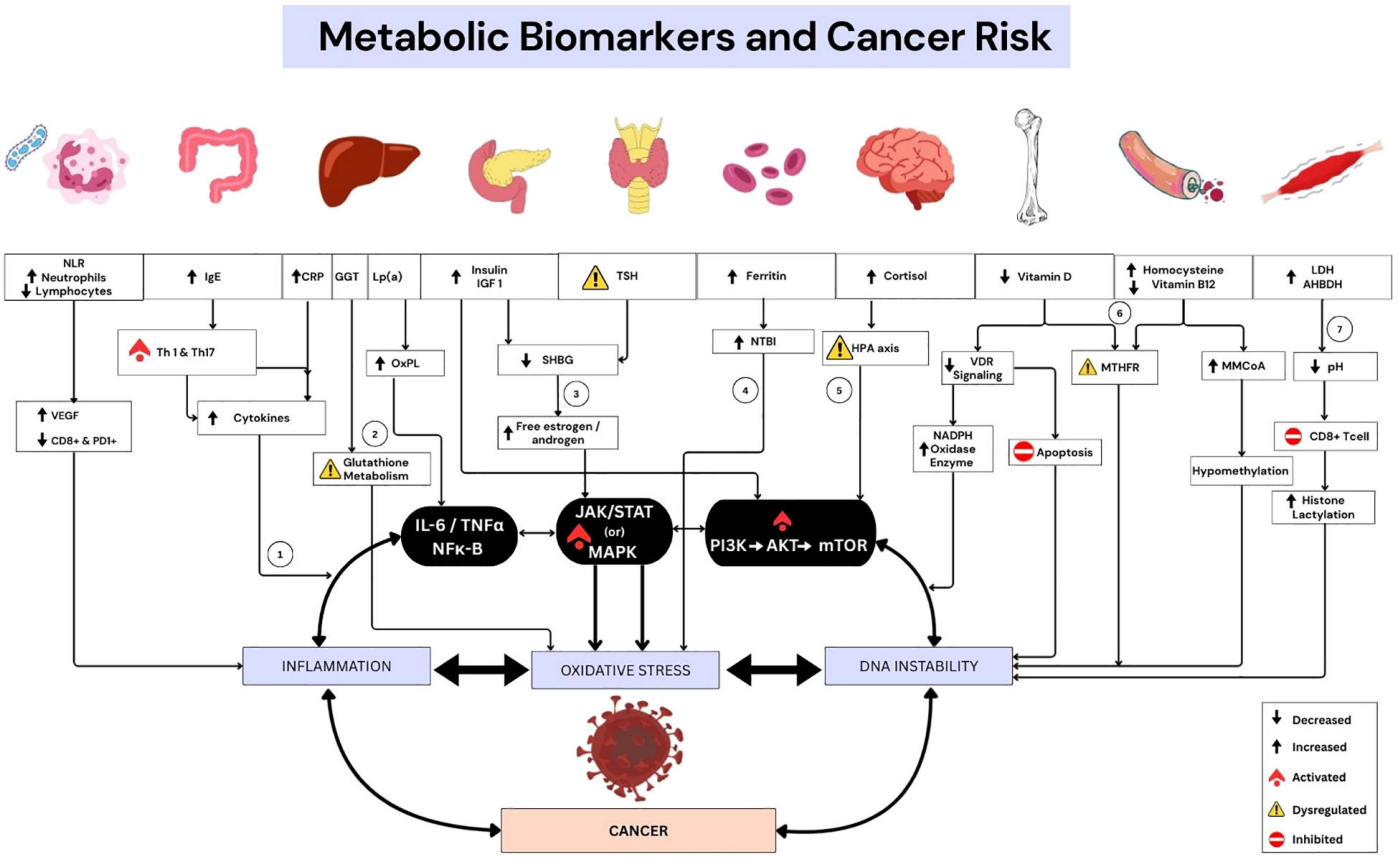
Metabolic biomarkers and cancer risk: An Overview of mechanisms. NLR, Neutrophil Lymphocyte Ratio; IgE , Immunoglobulin E; CRP, C-Reactive Protein; GGT , Gamma-Glutamyl Transferase; Lp(a) , Lipoprotein a; IGF 1 , Insulin -like Growth Factor 1; TSH , Thyroid Stimulating Hormone; LDH , Lactate Dehydrogenase; AHBDH – α-Hydroxybutyrate Dehydrogenas; Th , T helper cells (1 & 17); VEGF , Vascular Endothelial Growth Factor; OxPL - Oxidized Phospholipids; SHBG , Sex Hormone Binding Globulin; NTBI , Non-Transferrin Bond Iron; VDR , Vitamin-D Receptor; MTHFR , Methylenetetrahydrofolate reductase; MMCoA , Methylmalonyl Coenzyme A; HPA , Hypothalamic-Pituitary-Adrenal axis; IL6 , Interleukin 6; TNFα , Tumor Necrosis Factor α; NFκB , Nuclear Factor kappa B; JAK , Janus Kinase; STAT, Signal Transducer and Activator of Transcription; MAPK , Mitogen-Activated Protein Kinase; AKT , Protein Kinase B; PI3K , Phosphatidylinositol 3-kinase; mTOR , mechanistic Target of Rapamycin. 1. Immune imbalance: Altered NLR, IgE, and CRP drive NF-κB activation, VEGF release, and suppression of CD8^+^ immunity. 2. Metabolic stress: GGT reflects glutathione disruption, while Lp(a) delivers oxidized phospholipids fuelling systemic inflammation. 3. Growth signalling: Hyperinsulinemia with dysregulated TSH sustains PI3K–AKT–mTOR and MAPK pathways, reduces SHBG, elevates free sex hormones, and amplifies proliferative signaling. 4. Iron overload: Excess NTBI and ferritin generate ROS, intensifying oxidative injury. 5. Neuroendocrine stress: Cortisol dysregulates the HPA axis, maintaining high oxidative and inflammatory processes. 6. Vitamin and methylation axis: Low vitamin D downregulate VDR and links with MTHFR-driven DNA methylation and defective apoptosis; low B12 elevates homocysteine, compounding oxidative stress. 7. Acidic microenvironment: LDH-driven lactate acidifies tissues, inhibits cytotoxic T cells, skews macrophages, and induces histone lactylation, promoting neoplastic progression.

The selection and interpretation of metabolic markers discussed in this paper are grounded in the clinical insights and experiences gained from an integrative oncology clinic, where patient care merges conventional cancer treatments with metabolic and lifestyle-based interventions. Clinicians at the integrative oncology clinic routinely assess a spectrum of metabolic markers, not only to support ongoing cancer treatment, but also to detect early biochemical shifts that may signal elevated cancer risk. The metabolic markers discussed in this paper cannot be considered stand-alone indicators of cancer risk. Rather, their significance lies in the collective assessment of the interconnected biochemical pathways they influence (**Figure 1**). Disruptions in these pathways may act synergistically, creating a metabolic milieu conducive to abnormal genetic aberrations, impaired DNA repair, and uncontrolled cell proliferation. Therefore, integrating these markers into a broader panel of biochemical and molecular assessments could provide a more comprehensive risk evaluation framework, potentially improving early detection and preventive strategies.

The present study has identified several metabolic and biochemical markers consistently associated with cancer risk. This opens the possibility of developing a composite Metabolic Oncology Risk Index that integrates multiple metabolic biomarkers into a unified predictive framework. However, the present study did not examine the development of a composite Metabolic Oncology Risk Index, as this would require access to large, longitudinal datasets with standardized biomarker data, advanced statistical modelling for weight derivation, and benchmarking against established cancer risk tools. This represents a limitation of the current work, and future studies should address this gap by designing and validating such an integrative predictive framework to strengthen risk assessment in metabolic oncology.

Nevertheless, this proposal for early detection enables timely interventions aimed at restoring homeostasis and reducing the likelihood of malignant transformation. These markers can be considered actionable targets within individualized protocols that incorporate dietary therapy, mitochondrial support, stress reduction, and adjunctive therapies. By proactively addressing metabolic imbalances, this approach offers a valuable opportunity to mitigate disease progression and optimize therapeutic outcomes. Furthermore, it repositions metabolic biomarkers as dynamic tools not only for prognosis but also for prevention, integrating seamlessly with conventional oncology to establish a more anticipatory and patient-centered model of cancer care and prevention.

## Data Availability

The original contributions presented in the study are included in the article/**Supplementary Material**. Further inquiries can be directed to the corresponding author.
