# Supplementary material for: Decoding metabolic dysfunction in cancer: foundations for early detection and personalized therapeutics
Source: Front Endocrinol (Lausanne). 2025 Nov 24;16:1693142. doi: 10.3389/fendo.2025.1693142 (PMC12682692; doi:10.3389/fendo.2025.1693142)
Supplement: Supplementary file 1 [file Table1.docx]

**Metabolic Biomarkers and Cancer Risk**

| **Biomarker**  *(Assays)* | **Normal Range** | **Elevated/ Reduced Level & Cancer Risk** | **Major confounders to consider** | **Highest Level of evidence available supporting association** | **Summary of observations from literature** |
| --- | --- | --- | --- | --- | --- |
| **Ferritin**  *(Immunoassay-chemiluminescent)* | Men: 24–336 μg/L; Women: 11–307 μg/ | Elevated in cancers (breast, lung, ovarian, etc.) and correlates with tumor stage, excess iron promotes ROS/DNA damage[.^1^](https://www.mdpi.com/1467-3045/47/1/60#:~:text=iron%20contributes%20to%20the%20formation,Lipid%20peroxidation%20causes%20damage) | Inflammation, infection, iron overload disorders, liver disease, malignancy. | Meta-analysis and systematic review^2^ | Elevated serum ferritin is commonly observed in cancers such as pancreatic, renal, lung, and head and neck malignancies, serving as a sensitive marker of advanced stages, although one cohort study reported a contrary inverse association.^3^ |
| **Homocysteine (Hcy)**  *(HPLC or immunoassay)* | 5–15 μmol/L | Elevated in many cancers; high Hcy is associated with increased colorectal (and other) cancer risk, reflecting disturbed methylation pathways.^4^ | B12/folate status, renal function, medications | Meta-analysis^5^ | Elevated homocysteine (Hcy) levels have been linked to an increased overall risk of cancer, although a Mendelian randomization study found no significant association, particularly in breast, prostate, and renal cell carcinomas.^6^ |
| **Vitamin D (25(OH)D₃)**  *(LC-MS/MS (gold) or immunoassay)* | ~25–80 ng/mL (50–200 nmol/L) | Higher levels generally linked to lower risk of colorectal, breast, pancreatic cancers (strong inverse correlation).^7^  Deficiency (<20 ng/mL) is associated with higher incidence of several cancers (CRC, breast, etc.) ^7^ | Sun exposure, supplementation, BMI, season | Meta-analysis^8^ | Despite methodological and disease-specific differences, nearly all studies indicate a positive association between low vitamin D levels and an increased risk of cancer. |
| **LDH (Total)**  *(Enzymatic automated analyzers)* | ~140–280 U/L | Elevated in hypoxic tumors; high pre-treatment LDH strongly predicts worse survival across solid tumors. ^9^ | Hemolysis, liver disease, tissue injury | Meta-analysis and systematic review^10^ | Across studies LDH is reckoned as a metabolic and immune surveillance marker for both solid and hematologic tumors. |
| **HbA1c**  *(HPLC, immunoassay, enzymatic)* | <5.7% | Elevated HbA1c (even in non-diabetic range) is linked to higher cancer incidence across sites.^11^ | Diabetes, hemoglobin variants, anemia | Meta-analysis^12^ | There is consistent evidence across studies identifying HbA1C as an independent and modifiable predictor of cancer risk. |
| **Serum Insulin**  *(Immunoassay)* | Fasting: <25 µU/mL | Hyperinsulinemia drives the IGF-1 axis and mitogenic signaling; high fasting insulin associated with high cancer mortality.^13,14^ | Obesity, recent food intake, medications | Systematic review and meta-analysis^15^ | There is consistent evidence across studies indicating that serum insulin may serve as a potential biomarker for predicting cancer risk and prognosis. |
| **Cortisol** (AM)  *(Immunoassay; LC-MS for serum/saliva)* | ~10–20 μg/dL (06–10h) | Chronic elevated cortisol (stress-induced HPA-axis activation) causes immunosuppression and inflammation that can promote tumor growth.^16^ | Time of day, stress, glucocorticoid therapy, depression (mental health status) | Mendelian randomisation study^17^ | Despite variations across cancer sites, most of the studies suggest that elevated plasma cortisol levels may be associated with an increased risk of cancer. |
| **α-HBDH** (LDH isozyme)  *(Enzymatic assays)* | 95–220 U/L | Increased in malignancies (notably lung, testicular, breast cancers)^18–20^ | Hemolysis, myocardial/liver injury | Cohort studies^21,22^ | Most studies are observational in nature; however, consistent evidence suggests that elevated α-HBDH levels may serve as a risk factor for cancer. |
| **NLR**(Neutrophil:Lymphocyte)  *Automated CBC counters (ratio from counts)* | ~0.8–3.5 (varies by lab) | High NLR indicates systemic inflammation and immunosuppression; consistently predicts poorer prognosis in many cancers. ^23^ | Infection, systemic inflammation, corticosteroids | Meta-analysis^24^ | Across all studies, regardless of design or cancer type, NLR has been identified as a potential biomarker for predicting cancer risk and cancer-related outcomes. |
| **CRP** (C-reactive protein)  *(High-sensitivity immunoassay)* | <3 mg/L (normal) | Elevated CRP is associated with higher risk of breast, colorectal, lung, ovarian and other cancers.^25^ | Infection, obesity, smoking, chronic disease | Systematic review, prospective cohort and Mendelian randomization analysis^26,27^ | All studies explicitly highlight the diagnostic and prognostic significance of CRP in cancer. |
| **Vitamin B₁₂**  *(Immunoassay)* | ~180–914 pg/mL | Excess serum B₁₂ (>800–1000 pmol/L) is linked to increased risk of lung, pancreatic, liver, and myeloid cancers.^28^  B₁₂ deficiency leads to hyperhomocysteine and genomic instability, which can raise cancer risk.^29^ | Supplementation, liver disease, myeloproliferative disorders | Prospective cohort^30^ | Studies suggest that both elevated and deficient vitamin B12 levels have been reported as risk factors for cancer, except for one scoping review that found no temporal or causal relationship.^31^ |
| **IGF-1**  *(Immunoassay; LC-MS in research)* | *Age/sex-dependent (~100–350 ng/mL)* | High IGF-1 stimulates proliferation (via IGF-1R); elevated levels increase risk of breast, prostate, colorectal, melanoma, kidney, thyroid cancers[.^32^](https://pmc.ncbi.nlm.nih.gov/articles/PMC7642199/#:~:text=Participants%20were%20followed%20for%20a,CI%200.26%E2%80%930.38%29.%20The%20inverse)  Low IGF-1 is associated with higher risk of lung, ovarian, head & neck, and liver cancers (U-shaped effect)[.^32^](https://pmc.ncbi.nlm.nih.gov/articles/PMC7642199/#:~:text=Participants%20were%20followed%20for%20a,CI%200.26%E2%80%930.38%29.%20The%20inverse) | Age, sex, nutritional status, GH status | Case-control study^33,34^ | Most studies suggest that elevated IGF-1 levels are a risk factor for cancer, while a few report that reduced IGF-1 levels may also pose a risk. Additionally, some studies indicate no significant association between circulating IGF-1 levels and the risk of bladder cancer, lymphoma, or melanoma. |
| **Lipoprotein(a)**  *(Immunoassay)* | <30 mg/dL (<75 nmol/L) | High Lp(a) is an established cardiovascular risk (oncologic link unclear); some data suggest very high Lp(a) (especially with inflammation) may also relate to cancer mortality.^35^  Low Lp(a) (<80 mg/L in one study) was associated with ~1.5× higher cancer mortality (suggesting an anti-tumor role for Lp(a))^36^ | Genetic predisposition, lipid-lowering therapy, inflammation | Cohort study^37^ | The majority of population-based cohort studies suggest a strong association between elevated lipoprotein(a) levels and cancer risk; however, one large cohort study contradicts this finding. |
| **Total IgE**  *(Immunoassay)* | Adults: 0–100 IU/mL | Profound IgE deficiency (<2.5 kU/L) dramatically increases cancer odds (OR≈15) in children. ^38^  Elevated levels are associated with higher incidence of lung cancer and its poor prognosis.^39^ | Atopy, parasitic disease, immunodeficiency | Cohort study^39^ | Studies on IgE show conflicting results: some report that either IgE deficiency or elevated IgE levels may increase cancer risk, while certain epidemiological studies suggest that higher IgE levels may be protective against colorectal cancer, pancreatic cancer, and gliomas. |
| **Thyroid hormones (TSH, T4, T3)**  *(Immunoassay)* | TSH: 0.4–4.0 mIU/L  Free T4: 0.9–1.7 ng/dL  Free T3: 2.3–4.1 pg/mL | Hyperthyroid profile (low TSH, high T4/T3) is linked to higher risks of solid tumors (notably lung, breast, prostate)^40^  Hypothyroid profile (high TSH, low T4/T3) tends to lower cancer risk (relative protection)^40^ | Autoimmune thyroid disease, medications, iodine intake | Cohort study^41^ | All studies consistently link hyperthyroidism with an increased risk of cancer, whereas evidence regarding hypothyroidism is conflicting, with some studies suggesting a protective effect and others finding no association. |
| **GGT**  *(Enzymatic automated assays)* | <50 U/L (varies by lab) | Elevated GGT (liver oxidative stress marker) is strongly associated with metabolic syndrome and is linked to breast/lung/prostate cancers.^42^ | Alcohol use, liver disease, metabolic syndrome | Systematic review and meta-analysis^43^ | Evidence across studies consistently indicates a strong association between elevated GGT levels and cancer risk. |
| **PTH**  *(Immunoassay)* | 10–55 pg/mL | Primary hyperparathyroidism (elevated PTH) patients have ~28% higher overall cancer risk.^44^ | Primary hyperparathyroidism, vitamin D status, renal disease | Systematic review and meta-analysis^45^ | Across the available literature, a consistent pattern emerges indicating that elevated circulating parathyroid hormone (PTH) levels are associated with an increased risk of various cancers. |
| **Ionized Calcium (Ca²⁺)**  *(Ion-selective electrode)* | Adults: 4.5–5.6 mg/dL (1.05–1.3 mmol/L) | High ionized calcium is inversely associated with risk of cancer.^46^ | Albumin, pH, PTH, vitamin D status | Cohort study^46^ | Studies investigating the role of ionized calcium in cancer are limited; however, the available evidence generally supports an inverse relationship between ionized calcium levels and cancer risk. Notably, one prospective cohort study reported no significant association. |
| **Estrogen**  *(Immunoassay; LC-MS for low levels)* | Male- 10–50 pg/mL  Female(premenopausal)- 30–400 pg/mL  Female (postmenopausal)- 0–30 pg/mL | High estradiol is associated with higher cancer risk.^47^ | Menopausal status, HRT, adiposity, liver metabolism | Mendelian randomization study^48^ | All available evidence indicates a strong association between elevated estrogen levels and increased cancer risk, particularly in breast, lung, cervical, ovarian, and endometrial cancers. |
| **Progesterone**  *(Immunoassay; LC-MS for low levels)* | Male-Less than 1 ng/mL  Female (Premenopausal):  Pre-ovulation (Follicular phase) < 1 ng/mL  Mid-cycle (Luteal phase): 5 to 20 ng/mL  Female (Postmenopausal):<1 ng/mL | Chronic exogenous progestins (as in combined HRT) raise breast cancer risk.^49^  Low progesterone (anovulatory cycles or estrogen-only therapy) means “unopposed” estrogen, vastly increasing endometrial (and possibly breast) cancer risk.^50^ | Menstrual cycle phase, HRT, contraception, menopausal status | Case-cohort study^51^ | Elevated circulating progesterone levels have been associated with an increased risk of cancer. Most available evidence focuses on the role of progesterone in breast cancer, with a few studies exploring its involvement in prostate cancer. However, the limited data across other cancer types and sites restrict a comprehensive understanding of its broader oncogenic potential. |

**References**

1. Szymulewska-Konopko K, Reszeć-Giełażyn J, Małeczek M. Ferritin as an Effective Prognostic Factor and Potential Cancer Biomarker. *Current Issues in Molecular Biology 2025, Vol 47, Page 60*. 2025;47(1):60. doi:10.3390/CIMB47010060

2. Ramírez-Carmona W, Díaz-Fabregat B, Yuri Yoshigae A, et al. Are Serum Ferritin Levels a Reliable Cancer Biomarker? A Systematic Review and Meta-Analysis. *Nutr Cancer*. 2022;74(6):1917-1926. doi:10.1080/01635581.2021.1982996

3. Kim H, Han K, Ko SH, An HJ. Association between serum ferritin levels and colorectal cancer risk in Korea. *Korean J Intern Med*. 2022;37(6):1205. doi:10.3904/KJIM.2022.007

4. Shiao SPK, Lie A, Yu CH. Meta-analysis of homocysteine-related factors on the risk of colorectal cancer. *Oncotarget*. 2018;9(39):25681-25697. doi:10.18632/ONCOTARGET.25355,

5. Zhang D, Wen X, Wu W, Guo Y, Cui W. Elevated Homocysteine Level and Folate Deficiency Associated with Increased Overall Risk of Carcinogenesis: Meta-Analysis of 83 Case-Control Studies Involving 35,758 Individuals. *PLoS One*. 2015;10(5):e0123423. doi:10.1371/JOURNAL.PONE.0123423

6. He Q, Yang Z, Sun Y, et al. The Impact of Homocysteine on the Risk of Hormone-Related Cancers: A Mendelian Randomization Study. *Front Nutr*. 2021;8:645371. doi:10.3389/FNUT.2021.645371/FULL

7. Schömann-Finck M, Reichrath J. Umbrella Review on the Relationship between Vitamin D Levels and Cancer. *Nutrients 2024, Vol 16, Page 2720*. 2024;16(16):2720. doi:10.3390/NU16162720

8. Zhang L, Zou H, Zhao Y, et al. Association between blood circulating vitamin D and colorectal cancer risk in Asian countries: a systematic review and dose-response meta-analysis. *BMJ Open*. 2019;9(12):e030513. doi:10.1136/BMJOPEN-2019-030513

9. Farhana A, Lappin SL. Biochemistry, Lactate Dehydrogenase. *StatPearls*. Published online May 1, 2023. Accessed July 30, 2025. https://www.ncbi.nlm.nih.gov/books/NBK557536/

10. Chen J, Zou X. Prognostic significance of lactate dehydrogenase and its impact on the outcomes of gastric cancer: a systematic review and meta-analysis. *Front Oncol*. 2023;13:1247444. doi:10.3389/FONC.2023.1247444

11. Goto A, Noda M, Sawada N, et al. High hemoglobin A1c levels within the non‐diabetic range are associated with the risk of all cancers. *Int J Cancer*. 2015;138(7):1741. doi:10.1002/IJC.29917

12. Ling S, Sweeting M, Zaccardi F, Adlam D, Kadam UT. Glycosylated haemoglobin and prognosis in 10,536 people with cancer and pre-existing diabetes: a meta-analysis with dose-response analysis. *BMC Cancer*. 2022;22(1):1-12. doi:10.1186/S12885-022-10144-Y/FIGURES/3

13. Tsujimoto T, Kajio H, Sugiyama T. Association between hyperinsulinemia and increased risk of cancer death in nonobese and obese people: A population‐based observational study. *Int J Cancer*. 2017;141(1):102. doi:10.1002/IJC.30729

14. Ghanavati M, Rahmani J, Rinaldi G, Zand H. Fasting Insulin and Risk of Cancer Related Mortality in Non-diabetic Adults: A Dose-response Meta-analysis of Cohort Studies. *Curr Diabetes Rev*. 2019;16(4):357-363. doi:10.2174/1573399815666190906130544,

15. Màrmol JM, Carlsson M, Raun SH, et al. Insulin resistance in patients with cancer: a systematic review and meta-analysis. *Acta Oncol (Madr)*. 2023;62(4):364-371. doi:10.1080/0284186X.2023.2197124

16. Vignjević Petrinović S, Milošević MS, Marković D, Momčilović S. Interplay between stress and cancer—A focus on inflammation. *Front Physiol*. 2023;14:1119095. doi:10.3389/FPHYS.2023.1119095/BIBTEX

17. Larsson SC, Lee WH, Kar S, Burgess S, Allara E. Assessing the role of cortisol in cancer: a wide-ranged Mendelian randomisation study. *Br J Cancer*. 2021;125(7):1025. doi:10.1038/S41416-021-01505-8

18. Yuan ZM, Wang LH, Chen C. Prognostic value of serum α-HBDH levels in patients with lung cancer. *World J Surg Oncol*. 2023;21(1):78. doi:10.1186/S12957-023-02965-3

19. Ye J, Zheng L, Chen Z, et al. Serum α-hydroxybutyrate dehydrogenase as a biomarker for predicting survival outcomes in patients with UTUC after radical nephroureterectomy. *BMC Urol*. 2024;24(1):62. doi:10.1186/S12894-024-01439-2

20. Zhan CH, Liu GJ. Diagnostic Value of a Combined Serum α-Hydroxybutyrate Dehydrogenase, Carcinoembryonic Antigen and Glycoantigen 125 Test for Early-Stage Breast Cancer. *Breast Cancer: Targets and Therapy*. 2023;15:617-623. doi:10.2147/BCTT.S410500;WEBSITE:WEBSITE:TFOPB;PAGEGROUP:STRING:PUBLICATION

21. Yuan ZM, Wang LH, Chen C. Prognostic value of serum α-HBDH levels in patients with lung cancer. *World J Surg Oncol*. 2023;21(1):78. doi:10.1186/S12957-023-02965-3

22. Zhan CH, Liu GJ. Diagnostic Value of a Combined Serum α-Hydroxybutyrate Dehydrogenase, Carcinoembryonic Antigen and Glycoantigen 125 Test for Early-Stage Breast Cancer. *Breast Cancer: Targets and Therapy*. 2023;15:617-623. doi:10.2147/BCTT.S410500

23. Forget P, Khalifa C, Defour JP, Latinne D, Van Pel MC, De Kock M. What is the normal value of the neutrophil-to-lymphocyte ratio? *BMC Res Notes*. 2017;10(1):1-4. doi:10.1186/S13104-016-2335-5,

24. Feng J, Wang Y, Shan G, Gao L. Clinical and prognostic value of neutrophil-lymphocyte ratio for patients with thyroid cancer: A meta-analysis. *Medicine*. 2020;99(20):e19686. doi:10.1097/MD.0000000000019686

25. Guo L, Liu S, Zhang S, et al. C-reactive protein and risk of breast cancer: A systematic review and meta-analysis. *Sci Rep*. 2015;5(1):1-8. doi:10.1038/SREP10508;TECHMETA=139;SUBJMETA=1347,2423,499,53,67,692,699;KWRD=BREAST+CANCER,PREDICTIVE+MARKERS,RISK+FACTORS

26. Șiancu P, Oprinca GC, Vulcu AC, et al. The Significance of C-Reactive Protein Value and Tumor Grading for Malignant Tumors: A Systematic Review. *Diagnostics 2024, Vol 14, Page 2073*. 2024;14(18):2073. doi:10.3390/DIAGNOSTICS14182073

27. Zhu M, Ma Z, Zhang X, et al. C-reactive protein and cancer risk: a pan-cancer study of prospective cohort and Mendelian randomization analysis. *BMC Med*. 2022;20(1):301. doi:10.1186/S12916-022-02506-X

28. Amado-Garzon SB, Molina-Pimienta L, Vejarano-Pombo A, Vélez-Bonilla M, Moreno-Chaparro J, Buitrago-Lopez A. Elevated Vitamin B12, Risk of Cancer, and Mortality: A Systematic Review. *Cancer Invest*. 2024;42(6):515-526. doi:10.1080/07357907.2024.2366907,

29. Loedin AK, Speijer D. Is There a Carcinogenic Risk Attached to Vitamin B12 Deficient Diets and What Should We Do About It? Reviewing the Facts. *Mol Nutr Food Res*. 2021;65(6):2000945. doi:10.1002/MNFR.202000945

30. Arendt JFH, Sørensen HT, Horsfall LJ, Petersen I. Elevated vitamin B12 levels and cancer risk in UK primary care: A thin database cohort study. *Cancer Epidemiology Biomarkers and Prevention*. 2019;28(4):814-821. doi:10.1158/1055-9965.EPI-17-1136/70036/AM/ELEVATED-VITAMIN-B12-LEVELS-AND-CANCER-RISK-IN-UK

31. Obeid R. High Plasma Vitamin B12 and Cancer in Human Studies: A Scoping Review to Judge Causality and Alternative Explanations. *Nutrients*. 2022;14(21):4476. doi:10.3390/NU14214476/S1

32. Qian F, Huo D. Circulating Insulin-Like Growth Factor-1 and Risk of Total and 19 Site-Specific Cancers: Cohort Study Analyses from the UK Biobank. *Cancer Epidemiology Biomarkers and Prevention*. 2020;29(11):2332-2342. doi:10.1158/1055-9965.EPI-20-0743,

33. Adachi Y, Nojima M, Mori M, et al. Insulin-like growth factor-1, IGF binding protein-3, and the risk of esophageal cancer in a nested case-control study. *World J Gastroenterol*. 2017;23(19):3488-3495. doi:10.3748/wjg.v23.i19.3488

34. Price AJ, Allen NE, Appleby PN, et al. Insulin-like growth factor-I concentration and risk of prostate cancer: Results from the European prospective investigation into cancer and nutrition. *Cancer Epidemiology Biomarkers and Prevention*. 2012;21(9):1531-1541. doi:10.1158/1055-9965.EPI-12-0481-T/67247/AM/INSULIN-LIKE-GROWTH-FACTOR-I-CONCENTRATION-AND

35. Zhang Y, Wang W, Xu L, et al. Inflammation-Dependent Association of Lipoprotein (a) with Cardiovascular and Cancer Mortality. *Clin Epidemiol*. 2024;16:1. doi:10.2147/CLEP.S437456

36. Sawabe M, Tanaka N, Mieno MN, et al. Low lipoprotein(a) concentration is associated with cancer and all-cause deaths: A population-based cohort study (the jms cohort study). *PLoS One*. 2012;7(4). doi:10.1371/JOURNAL.PONE.0031954,

37. Sawabe M, Tanaka N, Mieno MN, et al. Low Lipoprotein(a) Concentration Is Associated with Cancer and All-Cause Deaths: A Population-Based Cohort Study (The JMS Cohort Study). *PLoS One*. 2012;7(4):e31954. doi:10.1371/JOURNAL.PONE.0031954

38. Uygun DFK, Uygun V, Başaran A, Kocatepe G, Kazlı T, Bingöl A. High malignancy rate in IgE-deficient children. *Int J Cancer*. 2025;156(5):964-968. doi:10.1002/IJC.35213,

39. Zhou D, Liu J, Zhang C, et al. Elevated serum total immunoglobulin E is associated with an increased risk of lung cancer: a retrospective study. *Front Immunol*. 2025;16:1637803. doi:10.3389/FIMMU.2025.1637803/BIBTEX

40. Khan SR, Chaker L, Ruiter R, et al. Thyroid function and cancer risk: The Rotterdam study. *Journal of Clinical Endocrinology and Metabolism*. 2016;101(12):5030-5036. doi:10.1210/JC.2016-2104,

41. Riis T, Bonnema SJ, Brix TH, Folkestad L. Hyperthyroidism and the risk of non-thyroid cancer: a Danish register-based long-term follow-up study. *Eur Thyroid J*. 2024;13(2). doi:10.1530/ETJ-23-0181

42. Ramandi A, George J, Behnoush AH, et al. The Association Between Serum Gamma-Glutamyl Transferase and Gastrointestinal Cancer Risk: A Systematic Review and Meta-Analysis. *Cancer Med*. 2025;14(2). doi:10.1002/CAM4.70581,

43. Ramandi A, George J, Behnoush AH, et al. The Association Between Serum Gamma‐Glutamyl Transferase and Gastrointestinal Cancer Risk: A Systematic Review and Meta‐Analysis. *Cancer Med*. 2025;14(2):e70581. doi:10.1002/CAM4.70581

44. Charoenngam N, Rittiphairoj T, Wannaphut C, Pangkanon W, Saowapa S. Risk of Malignant Neoplasm in Patients with Primary Hyperparathyroidism: A Systematic Review and Meta-analysis. *Calcif Tissue Int*. 2024;115(1):1-13. doi:10.1007/S00223-024-01219-Y/METRICS

45. Charoenngam N, Rittiphairoj T, Wannaphut C, Pangkanon W, Saowapa S. Risk of Malignant Neoplasm in Patients with Primary Hyperparathyroidism: A Systematic Review and Meta-analysis. *Calcif Tissue Int*. 2024;115(1):1-13. doi:10.1007/S00223-024-01219-Y/FIGURES/2

46. Karavasiloglou N, Hughes DJ, Murphy N, et al. Prediagnostic serum calcium concentrations and risk of colorectal cancer development in 2 large European prospective cohorts. *American Journal of Clinical Nutrition*. 2023;117(1):33-45. doi:10.1016/j.ajcnut.2022.10.004

47. Samavat H, Kurzer MS. Estrogen Metabolism and Breast Cancer. *Cancer Lett*. 2014;356(2 0 0):231. doi:10.1016/J.CANLET.2014.04.018

48. Johansson Å, Schmitz D, Höglund J, Hadizadeh F, Karlsson T, Ek WE. Investigating the Effect of Estradiol Levels on the Risk of Breast, Endometrial, and Ovarian Cancer. *J Endocr Soc*. 2022;6(8):1-9. doi:10.1210/JENDSO/BVAC100

49. Yue W, Wang J, Atkins KA, et al. Effect of a tissue selective estrogen complex on breast cancer: Role of unique properties of conjugated equine estrogen. *Int J Cancer*. 2018;143(5):1259. doi:10.1002/IJC.31401

50. Amiri M, Bidhendi-Yarandi R, Fallahzadeh A, Marzban Z, Tehrani FR. Risk of endometrial, ovarian, and breast cancers in women with polycystic ovary syndrome: A systematic review and meta-analysis. *Int J Reprod Biomed*. 2022;20(11):893. doi:10.18502/IJRM.V20I11.12357

51. Trabert B, Bauer DC, Buist DSM, et al. Association of Circulating Progesterone With Breast Cancer Risk Among Postmenopausal Women. *JAMA Netw Open*. 2020;3(4):e203645-e203645. doi:10.1001/JAMANETWORKOPEN.2020.3645
